# On Dissolving the Elastic Gum

**Published:** 1811-11

**Authors:** W. Hamilton

**Affiliations:** Surgeon. Ipswich


### *To the Editors of the Medical and Physical Journal.*

Gentlemen,

FINDING you mention, at the end of your last number, the request of a correspondent who signs himself Medicus, to be informed of the process for dissolving the elastic gum in æther, I have taken the liberty of sending you an account of the process as performed by Mr. Winch, apothecary, who, I believe, was the discoverer, and who transmitted a bottle of this solution from London to Macquer, at Paris. It is nearly as follows.

A pound of vitriolic æther is put into a bottle capable of containing about four pounds of any common liquid. On this æther there are poured two pounds of pure water, the bottle bottle is then stopped, held with the mouth downwards, and strongly shaken, in order to mix them. On discontinuing the agitation, the æther soon swims uppermost; the bottle is still held in the same position, and cautiously opened, keeping the thumb on the mouth of it. The water is by this means easily let off, and collected in a vessel below.

This operation is repeated two or three times with new quantities of water, until the sixteen ounces of æther are reduced to about five ounces. It is this purified remainder that is found to be the most perfect solvent of elastic gum, which is thrown into the æther after being cut into small pieces. It begins to swell in a very sbort time; the æther penetrates it, and appears to act slowly on it at first, but at the end of five or six hours, or later, the liquor is saturated and remains transparent. If there be a surplus of the gum it falls to the bottom, and on being removed from the bottle may be moulded into any form and will preserve its elasticity.

Having now described the manner of dissolving this curious substance, I shall add the most simple method of applying the part completely dissolved to use. A tube may be made in the following manner: Prepare a small cylinder of pipe-clay of the diameter and length of the intended tube. It is unnecessary to bake it, but sufficient merely to dry it. The æther saturated with the gum is poured into a glass or tin cylinder a little larger than the clay rod, and filled up to the brim with the said liquor. The operator then plunges the clay pipe into the æther, withdraws it suddenly and allows it to remain for an instant in the air, replunges it anew, and repeats the operation in proportion to the intended thickness of the tube, as each immersion leaves a small coating. The tube being thus formed of the elastic gum, is thrown into water in order to dissolve the clay-pipe. This the water very soon effects, and the gum is left in the form of a perfect tube.

By a similar process to the above, elastic bottles, &c. may be formed of any size or dimensions.

If you think the above worthy a place in your useful Journal, you are at liberty to insert it, and by so doing, satisfy the wishes of your correspondent, Medicus.

I remain, Gentlemen,

With great respect, and esteem,

Yours, &c.

W. HAMILTON, Surgeon.

